# Lysosomes in acute myeloid leukemia: potential therapeutic targets?

**DOI:** 10.1038/s41375-021-01388-x

**Published:** 2021-08-30

**Authors:** Sreoshee Rafiq, Sharon L. McKenna, Sylviane Muller, Mario P. Tschan, Magali Humbert

**Affiliations:** 1 Division of Experimental Pathology, Institute of Pathology, Bern, Switzerland; 2 Graduate School for Cellular and Biomedical Sciences, University of Bern, Bern, Switzerland; 3 Cancer Research, UCC, Western Gateway Building, University College Cork, Cork, Ireland; 4 TRANSAUTOPHAGY: European Network for Multidisciplinary Research and Translation of Autophagy Knowledge, COST Action CA15138, Barcelona, Spain; 5 CNRS and Strasbourg University Unit Biotechnology and Cell signaling / Strasbourg Drug Discovery and Development Institute (IMS); Ecole Supérieure de Biotechnologie de Strasbourg, Illkirch, France; 6 University of Strasbourg Institute for Advanced Study, Strasbourg, France

**Keywords:** Acute myeloid leukaemia, Drug development

## Abstract

Lysosomes, since their discovery, have been primarily known for degrading cellular macromolecules. However, in recent studies, they have begun to emerge as crucial regulators of cell homeostasis. They are at the crossroads of catabolic and anabolic pathways and are intricately involved in cellular trafficking, nutrient signaling, energy metabolism, and immune regulation. Their involvement in such essential cellular functions has renewed clinical interest in targeting the lysosome as a novel way to treat disease, particularly cancer. Acute myeloid leukemia (AML) is an aggressive blood cancer with a low survival probability, particularly in older patients. The genomic landscape of AML has been extensively characterized but few targeted therapies (with the exception of differentiation therapy) can achieve a long-term cure. Therefore, there is an unmet need for less intensive and more tolerable therapeutic interventions. In this review, we will give an overview on the myriad of functions performed by lysosomes and their importance in malignant disease. Furthermore, we will discuss their relevance in hematopoietic cells and different ways to potentially target them in AML.

## Introduction

### History and renewed interest

Lysosomes were first discovered by Christian de Duve in 1955 while working on a specific enzyme, glucose-6-phospatase, a target of insulin in hepatic tissue. Glucose-6-phosphatase was found to be encased in what was described as “sac-like particles” [1]. Several more pH sensitive hydrolases were then discovered in the same fraction of the cell and within a few years, the role of lysosomes in the digestion of extra- and intra-cellular material was established.

In the 1990s, the discovery of autophagy [2], generated a new appreciation for lysosomes as a degradative organelle critical for cellular recycling and homeostasis. However, as the final step in autophagic flux, lysosomes still lacked prestige due to their relegation to garbage disposal units. More recent studies have now elevated the lysosome to center stage with a new understanding of their role as a sensor of cell stress and coordinator of the response to a diverse range of environmental cues including nutrient, growth factor, and immune-related signaling [3, 4]. With that, clinical interest in the organelle for therapeutic intervention has also re-emerged [5].

In this review, we will focus on the key signaling functions of lysosomes, their potential role in the pathophysiology of malignant disease and consider why lysosomes might represent a good therapeutic target for acute myeloid leukemia (AML).

### Overview of lysosome function, biogenesis, and interaction with other organelles

Lysosomes are acidic organelles with an internal pH of 4.5 to 5.5, maintained by the presence of the vacuolar-type H^+ ^ATPase (V-ATPase) on the lysosomal membrane [6]. This pH range allows luminal hydrolytic enzymes, which degrade macromolecules, to function optimally. Lysosomes are heterogeneous in morphology, distribution, and function depending on the species and cell type. They can rapidly change their distribution, number, size, and activity to meet cellular needs. The formation of primary lysosomes requires fusion between endosomes and Golgi-derived vesicles containing hydrolytic enzymes tagged for lysosomal delivery [7].

The outer lysosomal membrane is rich in transmembrane proteins, predominantly lysosome-associated membrane proteins (LAMPs). Five types of LAMPs have been identified so far: LAMP-1/CD107a, LAMP-2/CD107b, LAMP-3/DC-LAMP, LAMP-4/Macrosialin/CD68 and LAMP-5/BAD-LAMP. LAMP-1 and LAMP-2 account for almost 80% of the lysosomal membrane proteins. Other key lysosomal membrane proteins (LMPs) include, ion channels and various receptors for specific cargo, tethering/fusion or signaling, such as the chloride channel protein 7 (CLC7), Niemann-Pick C1 protein (NPC1), synaptotagmin (SYT7), and the Vacuolar (V)-ATPase proton (H^+ ^) transporter [8].

Lysosomal genes are transcriptionally co-regulated in a network of genes referred to as the CLEAR network (Coordinated Lysosomal Expression and Regulation)—enabling a rapid response to metabolic demand. This gene network is regulated by transcription factors belonging to the microphthalmia/transcription factor E (MiT/TFE) family, including TFEB, TFE3, and MITF. They bind to a 10-bp palindromic motif sequence in the promoter sequence of lysosomal genes referred to as the CLEAR element. TFEB has also been shown to co-regulate the expression of several autophagy genes [9].

**Fig. 1:**
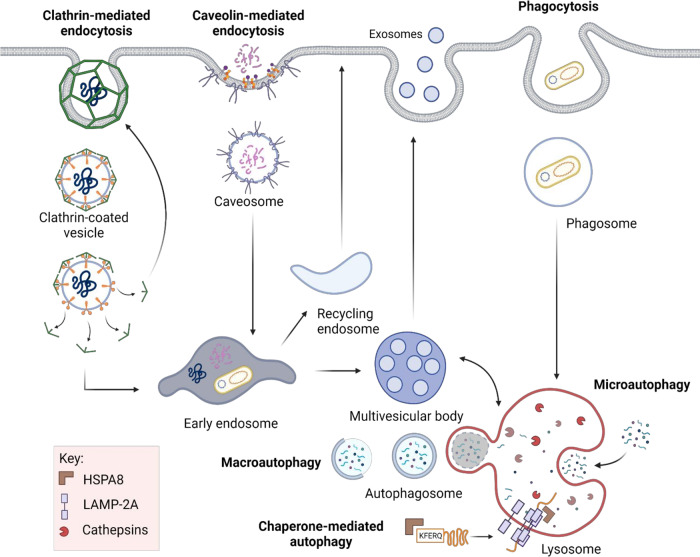
Main functions of lysosomes: Endosomal trafficking and autophagy pathways. Lysosomes are the terminal organelle for endocytic and autophagic pathways. External cargos are delivered to lysosomes via clathrin- and caveolin-dependent pathways or receptor-independent pathways such as phagocytosis. Late endosomes can either fuse with lysosomes, or they can release their contents outside the cell via extracellular vesicles known as exosomes. Cytoplasmic cargos are delivered to lysosomes for degradation by autophagosomes (macroautophagy), with the help of heat shock protein (HSP) A8 chaperone complex (CMA) or simply by invagination of the lysosomal membrane itself followed by engulfment (microautophagy). Created with BioRender.com.

Lysosomes are the terminal compartment for the delivery of cargo from clathrin- and caveolin-dependent and -independent endocytosis, autophagosomes, phagosomes, and chaperone-mediated autophagy (CMA) (Fig. 1). Interaction and fusion with other organelles are regulated by specific GTPases, Soluble N-éthylmaleimide-sensitive-factor Attachment protein REceptor (SNARE) complexes and Ca^2 + ^release from the lumen of the lysosome [10]. The primary role of lysosomal fusion is considered to be degradation of internal or external cargo into products that can be exported back to the cytoplasm for metabolic reuse, or in the case of professional antigen-presenting cells (APCs), presented as peptides by major histocompatibility complex (MHC) molecules to instigate an immune response [11]. However, fusion events, cargo components or contact with other ligands or organelles can also trigger signaling events [12].

### Signaling events arising from the intra-luminal compartment

The luminal compartment of lysosomes is the hydrolytic engine of the organelle, containing more than 60 acidic hydrolytic enzymes including proteases, nucleases, lipases, glycosidase phosphatases, and sulfatases. Deficiencies in any of these enzymes can have a major impact on cellular homeostasis. The lysosome is also a reservoir for important ions and metabolites including Ca^2 + ^/Fe^2 + ^/Zn^2 + ^, H^+ ^/Na^+^/K^+^, Cl^-^, and ATP. Intra-luminal levels of amino acids such as arginine and leucine are also regulated by lysosomal receptors and linked to external signaling [13].

#### Degradative enzymes and lysosome-dependent cell death

Cargo digestion is a key function of lysosomal enzymes. Several classes of enzymes will however also influence events outside the lysosome. Cathepsins are lysosomal proteases classed into, serine (A and G), aspartic (D and E), and cysteine proteases (B, C, F, H, K L, O, S, V, W, and X). The release of these proteases into the extracellular space has been associated with degradation of the extracellular matrix, cell migration, and invasion of cancer cells [14]. In addition, the release of proteases following lysosomal membrane leakage has been associated with cell death [15]. Several forms of lysosomal cell death (LCD) have now been described. Cells may exhibit necrotic, apoptotic, or apoptosis-like features depending on the extent of the leakage and the cellular context [16].

#### Lysosomes and calcium signaling

Lysosome membranes contain several ion channels that establish concentration gradients and maintain the lysosome membrane potential (Δψ_Lysol_). Relative to the cytoplasm, the lysosome has high concentrations of Ca^2 + ^, which is a key regulator of many lysosomal functions. There are at least three main types of Ca^2 + ^channels in mammalian lysosomes: Transient receptor potential mucolipin sub-family (TRPML)/mucolipin 1-3, (Two-Pore) TPC1-2 and P2X4. These respond to a variety of signals including cell stress, phospholipids, nutrients, and ATP depletion. The best-characterized channel is probably TRPML1/mucolipin 1, which regulates several lysosomal processes including lysosomal reformation, exocytosis, motility, and fusion with other organelles. Calcium efflux from TRPML1 also activates the calcium-dependent phosphatase calcineurin, which is important for TFEB nuclear translocation [17].

#### Luminal essential amino acid (EAA) sensing role

**Fig. 2:**
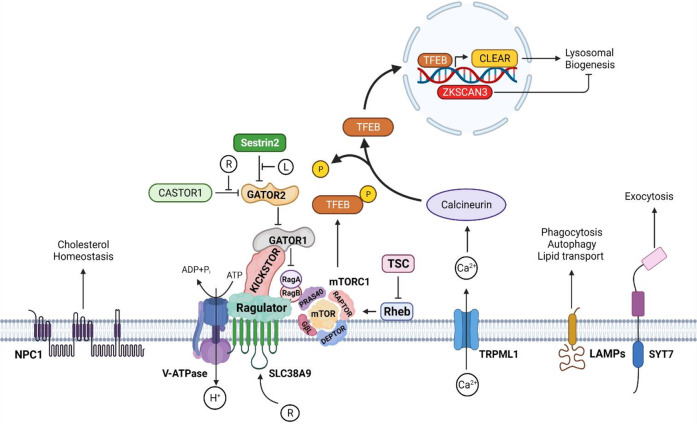
External and internal signaling events on the surface of lysosomes. Lysosomal membranes harbor a variety of proteins that regulate multiple signaling pathways. The transporter protein NPC1 releases free cholesterol from the lumen to maintain lipid homeostasis. The V-ATPase is a proton pump responsible for maintaining luminal pH. mTORC1 links metabolic functions to environmental signaling. The components of the mTORC1 signaling pathway can be regulated by both intra- and extra-luminal amino acid signaling. Arginine (R) in the lysosomal lumen is sensed by the amino acid transporter SLC38A9 which eventually activates mTORC1 with the help of the Rag GTPases and Ragulator complex. In the cytosol, Sestrin proteins serve as leucine (L) sensors and with the help of GATOR proteins, regulate mTORC1 activity. See text for further description. mTORC1 also negatively regulates lysosome biogenesis. Luminal Ca^2 + ^initiates lysosome biogenesis. It is released into the cytoplasm through the lysosomal calcium channel TRPML1 to activate calcineurin, which dephosphorylates TFEB, enabling it to translocate into the nucleus where it facilitates transcription of the CLEAR network genes. This pathway is inhibited by the transcription repressor ZKSCAN3. LAMPs are transmembrane proteins that account for about 80% of all membrane proteins and take part in various functions including, autophagy, lipid transport, and immune response. SYT7 is another calcium-dependent membrane protein that takes part in lysosomal exocytosis. Created with BioRender.com.

The lysosome can sense both luminal and cytosolic amino acid levels—with crosstalk between these pathways to maintain homeostasis or respond to nutrient-related signaling. Lysosomal luminal arginine is sensed by the sodium-coupled amino-acid transporter SLC38A9. The binding of arginine induces a conformational change in SLC38A9 leading to stimulation of the Rag A/B (Ras-related GTP-binding) GTPase and Ragulator complex localized on the lysosomal surface. This recruits the mechanistic target of rapamycin complex 1 (mTORC1), which is one of two protein kinase complexes incorporating the serine-threonine kinase mTOR. The mTORC1 complex consists of the mTOR kinase itself, Raptor, GβL, and DEPTOR. mTORC1 is then activated by Rheb (Ras homolog enriched in brain) GTPase, on the lysosomal membrane. Simultaneously, SLC38A9 also allows efflux of other amino acids such as leucine into the cytosol. This enables cross talk with other cytosolic amino acid sensors associated with mTORC1 complex (outlined below) [13, 18]. High levels of intra-luminal leucine can also activate mTORC1 by promoting ATP hydrolysis by the V-ATPase. This also stimulates the Ragulator/Rag complex to recruit mTORC1 [19] (Fig. 2).

### The lysosome surface as a signaling hub

As with other organelles, most intra-organelle communication and signaling is orchestrated at the surface. The lysosomal surface is a platform for the assembly of several signaling hubs that link metabolic functions to environmental signaling. A central link in this signaling is the mTORC1 complex. The primary role of active mTORC1 is to drive anabolic/biosynthetic pathways to fuel growth and proliferation and to inhibit catabolic pathways such as autophagy. Several signaling cues converge at mTORC1 including (i) nutrients (ii) growth factors and (iii) energy status.

#### Cytosolic amino acid signaling

Amino acids promote the translocation of mTORC1 to the lysosomal surface where it can be activated. This process is mediated by the coordinated actions of several complexes including Ragulator and Rag GTPases (A, B, C, and D). The Ragulator complex consists of 5 subunits; p18, p14, MP1, C7orf59, and HBXIP (also referred to as LAMTOR -1, 2, 3, 4, 5 respectively). The p18 subunit (LAMTOR-1) is anchored to the lysosomal membrane. In response to amino acids, Ragulator acts as a guanine nucleotide exchange factor (GEF) for Rag A/B—promoting their GTP bound state and anchoring both the Rag GTPases and mTORC1 to the membrane where it can be activated by the small GTPase Rheb [20, 21].

The presence of specific amino acid residues also influences other GEFs and GTPase-activating proteins (GAPs), upstream of GTP-RagA and GDP-RagC and mTORC1 signaling. A key integrator of amino acid signaling is the GTPase-activating proteins toward Rags (GATOR) complex [22]. GATOR is composed of two sub-complexes: GATOR1 and GATOR2. GATOR1 acts as a GAP (off switch) for RagA/B, releasing mTORC1 from the lysosome, thus acting as a negative regulator. GATOR2 positively regulates mTORC1 by inhibiting the GAP activity of GATOR1. Leucine and arginine residues act through stress-inducible proteins (Sestrins) and cytosolic arginine sensors for mTORC1 subunit 1 (CASTOR1) respectively. Upon amino acid binding, they dissociate from GATOR2, releasing their suppressive effects on GATOR2 and activate mTORC1 [23]. In the absence of amino acids, mTORC1 activity is also regulated by the cyclin-CDK inhibitor, cyclin-dependent kinase inhibitor 1C (CDKN1B) or p27. It binds to LAMTOR1 and prevents mTORC1 activation, which in turn, activates macroautophagy. In this context, p27 serves as a tumor suppressor [24].

#### Growth factors

Many external signals, such as growth factors converge at a large protein complex referred to as tuberous sclerosis complex (TSC), which negatively regulates the MTORC1 kinase [25]. TSC is a complex of TSC1, TSC2, and TBC1D7 subunits. TSC2 displays GTPase-activating protein (GAP) activity for Rheb, inactivating Rheb through induction of the GDP-bound state. Initially, signaling from receptors such as insulin or IGF (insulin-like growth factor) generates phosphatidylinositol lipids (3,4,5)P3 (PIP_3_), which stimulates protein kinase B (Akt) phosphorylation and inactivation of TSC2. This inactivation leads to an increase in GTP-loaded Rheb, which fuels activation of mTORC1 [26].

#### Energy status

In contrast to growth factor signaling, energy depletion activates AMP kinase at the lysosomal surface, which stimulates TSC2 through phosphorylation and inhibits RAPTOR. This leads to inhibition of mTORC1 and promotion of catabolic pathways [25].

### mTORC1 and transcriptional regulation of anabolic/catabolic pathways

mTORC1 also plays a major role in the phosphorylation of MiT/TFE factors. Under nutrient-rich conditions, mTORC1 phosphorylates TFEB on Ser 122 and Ser 211 and sequesters it to the cytoplasm. Inhibition of mTORC1 under a stressful condition, such as starvation, disrupts this association allowing nuclear translocation of TFEB and transcription of CLEAR network genes [3]. Another important regulator of TFEB nuclear translocation is the Ca^2 + ^-dependent phosphatase, calcineurin. During starvation, Ca^2 + ^is released through the lysosomal calcium channel, TRPML1/mucolipin-1, activating calcineurin, which dephosphorylates TFEB. A zinc finger family DNA-binding protein, ZKSCAN3, has been shown to be a transcriptional repressor of autophagy by repressing the expression of more than 60 essential genes associated with lysosomal biogenesis and autophagy including *Microtubule Associated Protein 1 Light Chain 3 Beta (Map1lc3b)* and *Wipi2*. ZKSCAN3 and TFEB have also been found to be oppositely regulated by starvation [27] (Fig. 2).

### Autophagy

Autophagy is a process by which cells transport dysfunctional or excessive cellular components into the lysosomes for degradation. Autophagy can eradicate harmful organelles, pathogens or aggregated material and the recycling of macromolecules from the lysosome enables cells to cope with stresses such as nutrient deficiency [28]. The three main types of autophagic pathways are macroautophagy, microautophagy and chaperone-mediated autophagy.

Macroautophagy is mediated by several protein conjugation reactions, eventually leading to the formation and expansion of a double-membraned autophagosome which carries the cargo to the lysosome to be degraded [29]. The genes involved in the process are known as ATG (AuTophaGy-related) genes, first identified in yeast, followed by studies of orthologues in other species [30]. In mammals, macroautophagy is initiated through activation of two key upstream regulatory complexes: (i) *The class III phosphatidylinositol 3-kinase* (*PI3K-III/ VPS34)* complex containing Beclin-1 (a mammalian homolog of yeast Atg6), hVps34, p150 (a mammalian homolog of yeast Vps15), and Atg14-like protein (Atg14L) and (ii) *the ULK1 kinase complex* (*ULK1-Atg13-FIP200-ATG101*)—this activates the class III PI3K/ Beclin-1 complex enabling autophagosome nucleation. These complexes are regulated by phosphorylation from the following pathways:

*The PI3K/AKT/mTOR signaling pathway:* Under abundant supply of nutrients and growth factors, cells activate the PI3K/AKT/mTOR signaling pathway and suppress autophagy, by inhibitory phosphorylation. In contrast, when cells are experiencing stress or nutrient starvation, they respond by inactivating the PI3K/AKT/mTOR pathway, which de-represses and activates autophagy.

*The AMPK signaling pathway:* AMPK (AMP-activated Protein Kinase) is a critical energy-sensing protein, which detects changes in the ratio of ATP to AMP or ADP. Phosphorylated AMPK phosphorylates TSC2 thereby inhibiting mTORC1 and activating autophagy. AMPK can also induce autophagy by directly phosphorylating and activating ULK-1/2. [31]

CMA is a client-specific, selective autophagic pathway. CMA clients contain a signature motif containing a short stretch of 5 amino acid residues, KFERQ (Lysine, Phenylalanine, Glutamate, Arginine, Glutamine) [32]. This motif is recognized by the chaperone protein HSPA8, also known as HSC70 or HSP73, which is a member of the HSP70 family of proteins [33]. The client protein is received on the lysosomal surface by a single molecule of LAMP-2A. LAMP-2A receptors form multimers that are stabilized by HSP90 on the lysosomal surface and aid translocation of the client. HSPA8 is also present inside the lysosome and helps in the translocation of the substrate by interacting with LAMP-2A [34].

In microautophagy, the dynamics of the lysosomal membrane allows it to wrap itself around cytosolic contents and engulf them into the lumen for degradation. Molecular details of microautophagy have not been elucidated as thoroughly as the other two pathways. Client specificity has been reported in endosomal microautophagy. Similar to CMA, the protein clients harbor a KFERQ-like motif and are delivered to the lysosomes by HSPA8 with the help of the endosomal sorting complex required for transport (ESCRT) machinery [35]. The ESCRT machinery helps to load protein into multivesicular bodies (MVBs), which are a special form of late endosome, that can then fuse with the lysosome [36].

### Subcellular localization of lysosomes

The signaling properties of lysosomes are affected by their position within the cell. Lysosomes can move from the center of the cell to the periphery or vice versa with the help of kinesin motors KIF2A and KIF1Bβ as well as the small GTPase ARL8B [37]. The consequences of lysosomal positioning seem to be dependent on the environmental signaling. Localization of lysosomes towards the periphery has been associated with increased interaction with mTORC1 and elevation of mTOR activity, whereas perinuclear localization in starved conditions inhibits mTORC1 and activates autophagy [38]. Hong and colleagues have shown that mechanistically, the FYVE-domain proteins Protrudin and FYVE and coiled-coil domain autophagy adaptor 1 (FYCO1) help to bring mTOR positive lysosomes towards the plasma membrane in a VPS34-dependent process [39]. Perinuclear clustering also delays mTORC2 reactivation in response to serum depletion [40]. In contrast, it has also been reported that under hypoxic conditions, lysosomes localize to the periphery and this actually reduces mTORC1 activity [41]. The tumor suppressor, folliculin is responsible for the association of lysosomes with the perinuclear membrane and restricting their localization in the area [42]. The subcellular localization of lysosomes also determines their luminal pH. Peripheral lysosomes are less acidic due to lowered V-ATPase activity [43]. The movement of lysosomes within the cell is essential for lysosomal exocytosis, which is a process that leads to the secretion of lysosomal content upon lysosome fusion with the plasma membrane. It is a ubiquitous, Ca^2 + ^-regulated mechanism, which plays a role in various physiological processes such as, plasma membrane repair and immunogenic ATP release [44].

### Galectins and lysophagy

The elimination of defective lysosomes by autophagy is referred to as ‘lysophagy’. Several β-galactoside-binding proteins (galectins) have been implicated in mediating lysophagy. GAL 3, 8 and 9 are recruited by exposure of luminal glycans following lysosomal damage. Galectin 8 can inhibit mTORC1 and Galectin 9 activates Mitogen-activated protein kinase kinase kinase 7 (TAK1 or MAP3K7) that stimulates AMPK [45]. Members of the galectin family have also been implicated in the control of differentiation and self-renewal of hematopoietic cells [46], and have been associated with prognosis in AML [47].

### Lipid homeostasis

Lysosomes are important regulators of lipid homeostasis and play a key role in cholesterol trafficking [48]. Low-density lipoproteins (LDLs) are trafficked to lysosomes via clathrin-mediated endocytosis. The release of free cholesterol from the lysosome is mediated by two transporter proteins, NPC 1 and 2 [49]. Lysosomes also contribute to maintaining cellular lipid balance through a selective autophagy pathway, referred to as lipophagy. Lipids are stored as triglycerides in lipid droplets (LD) which are delivered to lysosomes via lipophagy and degraded to release free fatty acids (FFAs) that can be used as an energy source [50].

### Immune responses/inflammation

Lysosomes can participate at several stages in the generation of an immune response. This includes pathogen sensing, phagocytosis, antigen processing and presentation, and inflammation/secretion.

Sentinel cells such as macrophages and dendritic cells (DCs) use toll-like receptors (TLR) to sense pathogens and elicit an immune response. Members of the TLR family, TLR 3 TLR7, TLR 8, and TLR9 have been shown to signal from endolysosomes. In addition, TLR9 responds to mitochondrial DNA, delivered to the lysosome via mitophagy [51, 52]. Foreign particles are internalized by phagosomes which eventually mature and fuse with lysosomes to become phagolysosomes. TFEB enhances phagocytosis in a calcium-dependent manner by transcriptional activation of immune-related genes [53]. Macrophages, DCs, and B cells are APCs that process antigens from degraded pathogens and incorporate them into MHCs on their surfaces. Lysosomal pH is important in this process as too much acidification results in excessive proteolysis of engulfed microbes, thus impairing cross-presentation. On the other hand, increased lysosomal pH can also alter lysosomal properties and impair antigen processing as seen in lupus, for example [54]. Lysosomes also participate in downregulation of inflammation in immune cells. The inflammasome complex, which is responsible for the secretion of pro-inflammatory cytokines such as IL-1β and IL-18, is selectively degraded via autophagy [55, 56]

## Lysosomes in malignancy

**Fig. 3:**
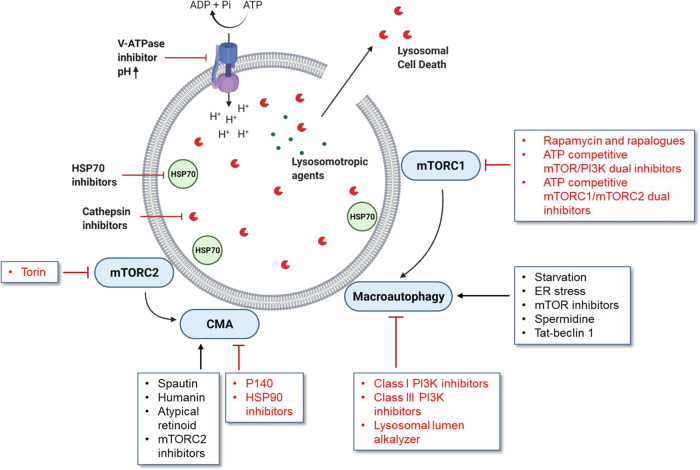
Various ways to modulate lysosomes and their functions. Lysosomes are associated with various pathways which makes it possible to target them in different ways. Lysosomotropic agents can disrupt the lysosomal membrane and release cathepsins into the cytosol leading to LCD. Other lysosomotropic agents such as chloroquine (or hydroxychloroquine) can impede lysosomal membrane fusion which is important for turnover in autophagy. Due to the protective role of HSP70 against lysosomotropic agents, HSP70 inhibitors can be used to intensify their effect. During oncogenic activation, lysosomal exocytosis causes release of cathepsins into extracellular space, which promotes extracellular matrix degradation and invasion of malignant cells. This makes protease inhibitors targeting cathepsins also a therapeutic option. Autophagic pathways have context-dependent roles in cancer and thus, there may be several strategies to either block or stimulate the pathways. Upstream pathways such as mTORC1 and 2 can be inhibited to stimulate autophagy in an indirect manner. V-ATPase inhibitors can elevate lysosomal pH, thus impairing the function of many pH-sensitive lysosomal enzymes. Created with BioRender.com.

Cell transformation and cancer progression require an increase in biomass production and adaptation to nutrient stress, processes that are aided by lysosomes. Accordingly, cancer cells have higher lysosomal activity compared to healthy adjacent tissue [57]. Lysosomes contribute to the maintenance of many hallmarks of cancer such as, sustaining proliferative signaling (mTORC1 signaling), metabolism (catabolic reactions, autophagy), and invasion (lysosomal exocytosis) [58]. For example, in sarcomas, downregulation of the protein NEU1 aggravates lysosomal exocytosis, which releases hydrolases and exosomes that facilitate extracellular matrix remodeling and invasion into the adjacent tissue [59]. Lysosomes are also known to sequester chemotherapeutic drugs and thus play an active role in the development of drug resistance [60]. In addition, lysosomes in metastatic cells differ greatly in content, localization, and activity from the lysosomes in normal cells which make them potentially more susceptible to lysosome targeting agents [61]. Since lysosomes are central to many cellular pathways, several ways exist to target them indirectly for cancer therapy (Fig. 3).

Direct impairment of lysosomal function can be achieved through several strategies. Most of the therapeutic agents being developed to target lysosomes either raise the luminal pH to inactivate cathepsins thus rendering them unable to mediate invasion, or aid in lysosomal membrane permeabilization and induce LCD. Lysosome targeting agents and the diseases they are being tested against has already been adequately reviewed [62]. In Table 1, we have summarized the mechanism of a few emerging preclinical options which can affect lysosomes, the diseases they have been tested with, and their toxicity profile.

**Table 1:** Examples of agents that can target lysosome structure and functions in cancers.

Drug	Type	Disease	Mechanism	Toxicity/ specificity	References
Hydroxychloroquine	Lysosomotropic agent	Various solid tumors	Sequestered within the lysosome, raising pH resulting in non-functional cathepsins	Acute toxicity is rarely observed in typical use. High dose and prolonged use can affect several organ systems	[105]
		Lymphoma	Impedes autophagic flux		[106]
		Myeloma			
Minnelide/Triptolide	Triptolide analogue (water soluble)	Colon cancer	Induces lysosome-mediated programmed cell death	Affects several signaling pathways and induce hepatotoxicity, cardiotoxicity, reproductive toxicity etc.	[107]
		Breast cancer			[108]
		Non-small-cell lung carcinoma (NSCLC)			[109]
		AML			[110]
					[111]
Mixed-charge nanoparticles	pH dependent aggregator	Breast cancer	Crystallize inside cancer lysosomes, impair their function and induce lysosome-dependent cell death	No clinical information available yet	[112]
		Lung cancer			
		Melanoma			
Saliphenylhalamide	Vacuolar ATPase inhibitor	Breast cancer	Impaired lysosomal acidification	Have immune-modulating properties. Insufficient in vivo data	[113]
		Lung cancer			[114]
					[115]
					[116]
VER-155008	HSP70 inhibitor	Colon carcinoma	Aids in lysosomal membrane permeabilization	Low bioavailability	[117]
					[118]
WX8, Apilimod	Phosphoinositide Kinase, FYVE-Type Zinc Finger Containing (PIKfyve) inhibitor	B-cell malignancies	Disruption of lysosomal homeostasis by enlargement of lysosomes, impairment of trafficking, and prevention of autophagic cargo degradation	Concentration required for anti-cancer effect also inhibits proliferation in normal cells resulting in altered immune response and insulin sensitivity in patients.	[119]
		Melanoma			[120]

## Lysosomes in AML

Hematopoiesis is a complex process that gives rise to cells with strikingly distinct morphology and functions. Such processes require drastic reorganization of cellular homeostasis. Lysosomes play a vital role here as they maintain a balance between anabolic and catabolic pathways. Due to their association with the master regulator of cell growth, mTORC1, and the ability to degrade macromolecules via autophagic pathways, they are in a central position to modulate metabolism, differentiation, proliferation, and cell death. All of these components are essential in the maintenance of functioning hematopoiesis and their dysregulation can be a feature of hematological malignancies.

### Lysosome derived structures in myeloid leukemia cells

**Fig. 4:**
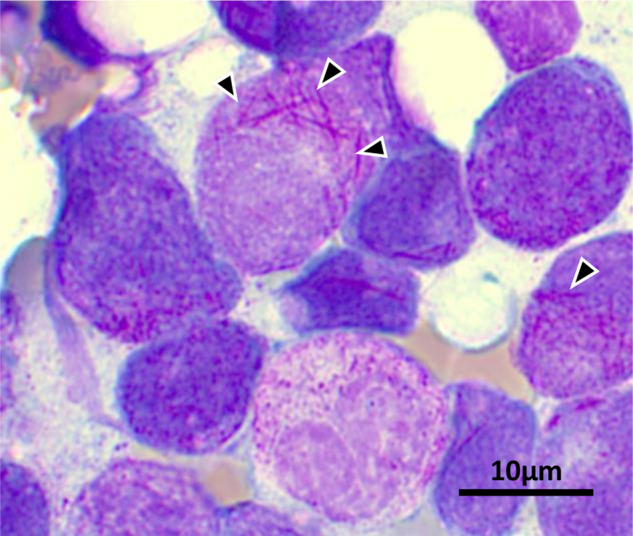
Auer Rods. Image of acute promyelocytic leukemia (APL) blast cell. Black arrow heads indicate bundles of needle-shaped Auer rods. See text for further description. (Courtesy: Myriam Legros, Center of Laboratory Medicine, University Hospital Bern). Informed consent is available for all patients in oncology.

Auer rods (or Auer bodies) are large structures sometimes observed in AML, acute promyelocytic leukemia (APL), and myelodysplastic syndromes (Fig. 4). Auer rods are azurophilic, can resemble needles, commas, diamonds, rectangles, corkscrews, and can appear bundled. They are not always present—but are a highly specific finding and virtually pathognomonic for a myeloid neoplastic disorder. They are apparently composed of fused lysosomes and are rich in lysosomal enzymes. Their method of formation and functional significance are not known but several processes have been described that cause tubulation of lysosomes including autophagic lysosome reformation (ALR) [63] and activation of antigen-presenting cells. Activation of macrophages and DCs causes dramatic tubulation of endolysosomes—which increases their surface area to volume ratio and is also proposed to assist with the delivery of MHC-II peptides to the cell surface for presentation [64].

In positive AML cases, the percentage of cells with Auer rods and the number of Auer rods per cell can vary greatly. Auer rods are found in AML M1, M2, M3, M4, M5, and M6, but not in AML M0 or M7 of the French–American–British (FAB) classification, or in blast crisis chronic myeloid leukemia, suggesting an association with a more mature myeloid phenotype. While Auer rods clearly have diagnostic value in determining the neoplastic nature of myeloid cells, they do not seem to have a clear prognostic significance [65].

### Lysosomes in hematopoietic stem cells (HSC)

HSC are responsible for repopulating blood cells and maintaining blood homeostasis. Therefore, in order to protect themselves from stress, they usually remain quiescent. Liang et al., reported that, quiescent HSCs are defined by their low mitochondrial membrane potential and glycolysis. Enlarged lysosomes are found in quiescent HSCs, which are speculated to remove toxic products and contribute to the maintenance of healthy HSCs [66]. Lysosomes are asymmetrically inherited by the daughter cells during cell division of HSCs and therefore determine their cellular fate by reorganizing the metabolic and translational machinery of the cells [67].

### Autophagy in HSCs

Autophagic pathways, in particular, macroautophagy contributes to the maintenance of energy metabolism, which is crucial in HSCs. The transcription factor FOXO3A, drives a gene expression program that induces autophagy to protect HSCs during stress [68]. The specific knockout of *Atg7* in the hematopoietic system of mice results in severe clinical symptoms (lethargy, piloerection, and weight loss), leading to their death within 12 weeks. The *Atg7*^*−/−*^ LSK (Lin^−^ Sca-1^+^ Kit^+^) cells showed accumulation of mitochondria and DNA damage with complete loss of stem cell function [69]. Similar pre-leukemic phenotypes were observed in *Atg5* knockout mice [70]. FIP200, a key protein in the autophagosome nucleation process, is necessary for fetal HSC maintenance and its loss leads to a block in erythroid maturation, depletion, and loss of reconstituting capacity of HSCs, and aberrant expansion of myeloid cells [71]. A recent study reported that CMA is crucial for protein quality control and metabolic reprogramming during HSC activation. It also maintains the functionality of aged HSCs [72].

### Autophagy in AML

Autophagy dysregulation can lead to and drive AML. An in silico analysis has shown that a deleted chromosomal region often found in AML coincides with the location of autophagy genes and that lowered autophagic flux is advantageous to AML [70]. Heterozygous deletion of *Atg5* in Mixed Lineage Leukemia-Eleven Nineteen Leukemia (MLL-ENL) induced murine HSCs led to a higher proliferation rate and a more aggressive leukemia in vivo due to a shift towards glycolytic metabolism [70]. Depletion of ATG7 was found to enhance chemoresistance in mice transplanted with OCI-AML3 cells harboring *Atg7* targeting shRNA [73]. In a MLL-AF9 driven model, deletion of *Atg5* accelerated the initiation of leukemia but had no effect on therapeutic intervention [74]. Conversely, another study on MLL-ENL leukemic mice showed that homozygous deletion of *Atg5* or *Atg7* in bone marrow cells reduced the frequency of leukemia-initiating cells (LICs) and decelerated leukemia progression [75]. In FLT3-ITD driven AML, the receptor tyrosine kinase, RET, has been identified as an essential driver of leukemogenesis. Mechanistically, it does so by activating mTORC1, thus, suppressing the ability of autophagy to degrade the FLT3 protein [76]. In contrast, another group found that FLT3-ITD upregulates basal autophagy mediated by the transcription factor, ATF4 and inhibition of autophagy could in fact, facilitate increased survival of mice with FLT3-ITD driven AML [77]. The aforementioned examples aptly demonstrate that, while the relevance of autophagy in AML is undeniable, inconsistencies between results exist possibly because the process has different implications at different levels of leukemic transformation.

### Autophagy in myeloid differentiation

Several studies indicate that autophagy plays a key role in myeloid differentiation. Macroautophagy is upregulated during colony-stimulating factor-1 mediated monocyte-macrophage differentiation and is necessary for cell survival as it can direct cells towards differentiation in lieu of apoptosis [78, 79]. Mechanistically, it has been shown that autophagy-mediated lipolysis is necessary for neutrophil differentiation [80].

Granulocytic differentiation can be induced in the AML subtype APL by treatment with all-*trans* retinoic acid (ATRA). Autophagy can facilitate degradation of the PML-RARA onco-protein in APL, but it is also important for differentiation of AML cells that do not have a fusion protein [81, 82]. ATRA-induced differentiation and autophagy were found to be dependent upon upregulation of TFEB [83]. More recently, it was shown that degradation of fatty acid synthase via ATRA-induced macroautophagy activates lysosomal biogenesis and accelerates granulocytic differentiation [84].

## Targeting lysosomes in AML therapy

**Fig. 5:**
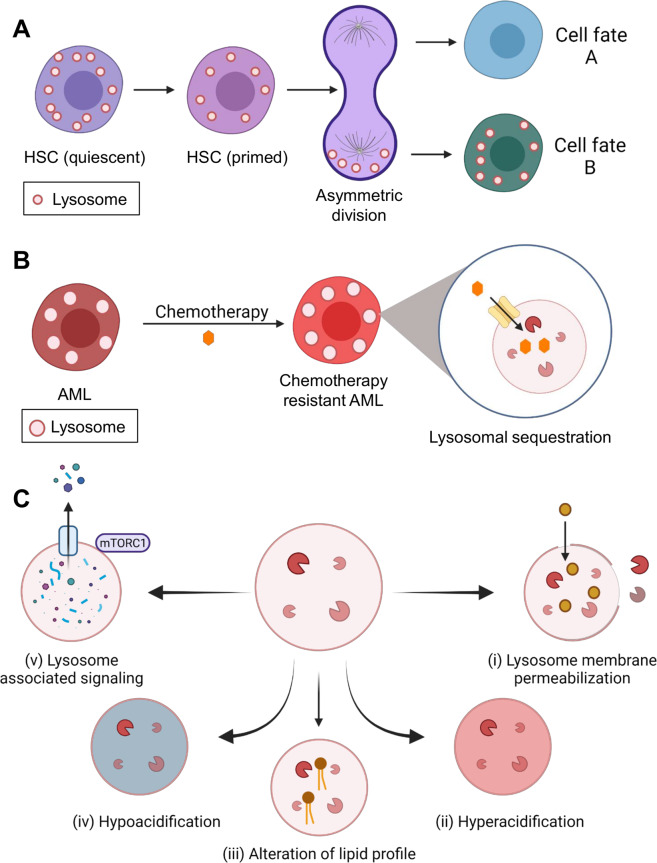
Role of lysosomes in hematopoiesis and AML. **A** Lysosomes play important roles in HSC maintenance and during cell division their asymmetric inheritance can influence cell fate. **B** Lysosomes are enlarged in AML cells and these can sequester therapeutic drugs to promote chemoresistance. **C** The various agents designed to target lysosomes in AML lead to five main consequences: (i) disruption of lysosome membranes, leading to release of luminal enzymes into the cytosol to cause LCD, (ii & iv) hyper- or hypo-acidification of the lysosomal lumen which disrupts lysosome function and can increase susceptibility towards certain drugs, (iii) alteration of luminal lipid profiles leading to lysosome-dependent cell death, (iv) blocking activity of pumps or channels such as the vacuolar-type H^+ ^ATPase to reduce luminal acidity or impair other lysosomal homeostasis, and (v) targeting various signaling proteins on the membrane surface to functionally impair lysosomes. Created with BioRender.com.

The lysosome is clearly a multi-functional organelle and there may be many ways to target it for therapeutic gain in leukemia. Moreover, lysosomes in AML cells are bigger in size which could make AML cells more susceptible towards lysosomal disruption than normal cells [85]. Several studies have investigated areas such as: direct structural damage, interference with pH or other luminal homeostasis, blocking associated signaling or interference with key trafficking events (Fig. 5). Similar to solid cancers, lysosomes also sequester chemotherapeutic drugs and confer resistance in AML [86]. Therefore, targeting lysosomes can not only serve as a way to destroy AML cells while sparing normal hematopoietic cells, but may also be a strategy to combat chemotherapy resistance.

### Structural damage

In 1994, Craig Rosenfeld demonstrated the antileukemic property of the lysosomotropic compound phenylalanine methyl ester in leukemic specimens derived from patients [87].

A screen of a library of 100 drugs showed that the anti-malarial drug, mefloquine, is effective against AML cell lines as well as leukemia patient samples. A gene set enrichment analysis carried out in yeast identified genes associated with lysosomal functions to be enriched in gene deletion strains that were sensitive to mefloquine, indicating that mefloquine targets lysosomes. Mefloquine disrupts the lysosomal membrane of AML cells allowing the release of cathepsins B and L, which results in cell death [85]. Another in silico screening assay of FDA-approved drugs identified a distinct subset of cationic-amphiphilic antihistamines that selectively killed AML cell lines and primary patient samples. Their toxicity was associated with the simultaneous disruption of both lysosomes and mitochondria which triggered both autophagy and apoptosis [88]. High expression of p53 induced gene 7 (*pig7)*, which encodes for the small integral membrane protein of lysosome/late endosome (SIMPLE) was found to induce lysosomal membrane permeabilization in AML cell lines and render them more susceptible to chemotherapies such as VP16 and daunorubicin [89].

### Interference with pH or other aspects of luminal homeostasis

The compound, deoxysappanone B 7,4′-dimethyl ether (Deox B 7,4) was identified from a chemical screen of small molecules with anti-leukemic activity. It is a microtubule inhibitor, which indirectly increases lysosomal V-ATPase activity causing hyper-acidification. This effect leads to lysosomal disruption and the death of AML cells. Interestingly, AML cells were found to be more vulnerable to Deox B 7,4, compared to normal hematopoietic cells [90]. Conversely, blocking lysosomal acidification via the V-ATPase inhibitor Archazolid A also has anti-leukemic effect. Archazolid A reduces the anti-apoptotic protein survivin which leads to cell death [91]. Several small molecules known as cationic amphiphilic drugs (CAD) are already used in the treatment of various disorders and possess lysosomotropic properties [92]. CADs are defined structurally by the presence of both a hydrophobic and hydrophilic domain. Many antihistamines, antidepressents, and even mefloquine are classified as CADs. A recent study has reported that CADs can induce lysosome-dependent cell death in various AML cell lines by altering the lipid profile within the lysosomal lumen. The compounds tested were the following antihistamines: desloratadine, ebastine, loratadine, astemizole, and terfenadine; the antimalarials: chloroquine and mefloquine; and the antidepressants: desipramine, penfluridol, and siramesine, among which siramesine and terfenadine had the strongest effects [93]. The flavonoid polyphenol, quercetin has also been reported to induce lysosome-dependent cell death in leukemia and is effective against multi-drug resistant HL60 cell lines [94].

### Lysosomal associated signaling

Several mTORC1 inhibitors, including rapamycin and its analogous (rapalogs) have been developed. Many second-generation inhibitors have also been developed that target both mTORC1 and mTORC2, or mTORC1 and PI3K. Phase I/II studies with rapalogs as single agents in AML have been disappointing, whereas combination studies with chemotherapy have demonstrated some promise [95]. Interestingly, the mTORC1/2 dual inhibitor AZD2014 was found to reduce the pH of lysosomes in AML cells and this enhanced the cytotoxicity of the antibody-drug conjugate Gemtuzumab ozogamicin (GO) [96]. The use of an antibody conjugate such as GO in AML is particularly appealing as most AMLs express CD33. GO requires an acidic environment inside lysosomes for hydrolysis of the linker molecule and cytocidal activity. The mTORC1-associated protein, raptor, represents a potential target in AML, as its ablation blocks leukemia progression. However, it does not affect leukemic stem cell (LSC) renewal [97]. mTORC1 inhibition sensitizes the cells to lysine-specific demethylase 1 inhibitors and triggers differentiation in MLL leukemia [98]. The lysosomal membrane protein LAMP5 acts as an autophagy suppressor and targeting it promotes autophagic degradation of the MLL fusion protein, which extends survival [99]. A novel ATRA derivative, 4-amino-2-trifluoromethyl-phenyl retinate (ATPR) has demonstrated superior anti-cancer efficacy against AML by inducing ferroptosis in AML cells through a mechanism involving macroautophagy [100].

### Other novel approaches

An innovative approach to target AML cells involved the development of a biohybrid with a tumor-associated peptide somatostatin and the photosensitizer ruthenium, named RU-SST. The stomatostatin receptor type 2 (SSRT2) is expressed more highly on AML cell lines and leukemic cells of patients with AML compared to HSCs from healthy donors. Therefore, the biohybrid more specifically targets AML cells. Interestingly, RU-SST localizes within the lysosomes, indicating their involvement in the eradication mechanism [101]. Non-thermal plasma (NTP) is a relatively new addition in the field of cancer therapy. It is a form of ionized gas consisting of atoms and molecules in an excited state. NTP has been shown to induce cell death in AML cell lines, HL60, and KG-1 via lysosome inhibition [102].

Lysosomes can be utilized in a rather unique manner by harnessing them as a biologically derived nano-carrier. Encapsulation of cancer drugs within nano-carriers such as liposomes has already been put into practice. Lysosomes, due to their robustness within the biological environment and low immunogenic reaction can serve as a suitable carrier. Yeast-derived lysosomes, engineered to carry the anthracycline drug, daunorubicin, have exhibited efficacy against the AML cell line, HL60 [103].

## Conclusion and perspectives

This review highlights the variety of functions associated with lysosomes and emphasizes their central role in maintaining cellular homeostasis. It is hardly surprising that deregulation of lysosomal structure and function can play a key role in malignant diseases such as AML. Tumor-specific lysosomal alterations have been identified, opening the possibility of targeting lysosomes as a way to exert maximum effect on cancer cells while sparing normal ones. To date, the cornerstone of chemotherapy treatment in AML is the combination of cytarabine (Ara-C) with an anthracycline and/or allogeneic stem cell transplantation. However, patients above the age of 65 respond poorly to such a taxing treatment regime. On the other hand, targeted therapies, while less strenuous, are usually not effective as a monotherapy [104]. In the scope of this review, we have highlighted lysosomal functions that may be targeted therapeutically and potentially convey a synergistic effect when combined with other modes of targeted therapies.

One major issue in the field of lysosomal research is that the lysosome has become synonymous with its degradative functions and more attention needs to be drawn towards the lysosome as a signaling organelle. Importantly, targeting the lysosome is independent of the molecular lesions in AML and is not dependent upon the disruption of up-stream receptor-mediated signaling events—for which resistance is inevitable. New targeting or chemo-sensitizing strategies of this type would therefore be extremely valuable. We hope that this review reiterates the importance of studying lysosomes and reinforces the growing interest in exploring its value as a therapeutic target in leukemia.
